# Personalisation of Plantarflexor Musculotendon Model Parameters in Children with Cerebral Palsy

**DOI:** 10.1007/s10439-022-03107-8

**Published:** 2022-11-15

**Authors:** Kirsten Veerkamp, Marjolein M. van der Krogt, Jaap Harlaar, Thomas D. O’Brien, Barbara Kalkman, Ajay Seth, Lynn Bar-On

**Affiliations:** 1 Department of Rehabilitation Medicine, Amsterdam UMC, Vrije Universiteit Amsterdam, Boelelaan 1117, Amsterdam, The Netherlands; 2 Amsterdam Movement Sciences, Rehabilitation & Development, Amsterdam, The Netherlands; 3 School of Health Sciences and Social Work, Griffith University, Gold Coast, Australia; 4 Griffith Centre of Biomedical & Rehabilitation Engineering (GCORE), Menzies Health Institute Queensland, and Advanced Design and Prototyping Technologies Institute (ADAPT), Griffith University, Gold Coast, Australia; 5 Department of Biomechanical Engineering, Delft University of Technology, Delft, The Netherlands; 6 Department of Orthopedics & Sports Medicine, Erasmus Medical Center, Rotterdam, The Netherlands; 7 Research Institute for Sport and Exercise Science, Liverpool John Moores University, Liverpool, UK; 8 Department of Rehabilitation Sciences, Ghent University, Ghent, Belgium

**Keywords:** OpenSim, Neuromusculoskeletal modelling, Subject specific, Contracture, Ultrasound, Achilles tendon, Calf muscles, Triceps surae, Muscle mechanics, Biomechanical simulation

## Abstract

Neuromusculoskeletal models can be used to evaluate aberrant muscle function in cerebral palsy (CP), for example by estimating muscle and joint contact forces during gait. However, to be accurate, models should include representative musculotendon parameters. We aimed to estimate personalised parameters that capture the mechanical behaviour of the plantarflexors in children with CP and typically developing (TD) children. Ankle angle (using motion capture), torque (using a load-cell), and medial gastrocnemius fascicle lengths (using ultrasound) were measured during slow passive ankle dorsiflexion rotation for thirteen children with spastic CP and thirteen TD children. Per subject, the measured rotation was input to a scaled OpenSim model to simulate the torque and fascicle length output. Musculotendon model parameters were personalised by the best match between simulated and experimental torque–angle and fascicle length-angle curves according to a least-squares fit. Personalised tendon slack lengths were significantly longer and optimal fibre lengths significantly shorter in CP than model defaults and than in TD. Personalised tendon compliance was substantially higher in both groups compared to the model default. The presented method to personalise musculotendon parameters will likely yield more accurate simulations of subject-specific muscle mechanics, to help us understand the effects of altered musculotendon properties in CP.

**Supplementary Information:**

The online version contains supplementary material available at 10.1007/s10439-022-03107-8.

## Introduction

Cerebral palsy (CP) is a motor disorder attributed to lesions in the infant brain.^39^ Due to both neural and musculoskeletal adaptations, children with CP often develop gait pathologies.^19^ Particularly, limited ankle range of motion compared to typically developing (TD) children reduces gait efficiency.^6^ To gain insights into the underlying causes of limited ankle motion, previous studies have combined torque and ultrasound measurements during a slow passive stretch of the ankle^7,22,25,41,49^ to identify morphological and mechanical changes affecting muscle function. These studies have demonstrated mechanical adaptations of calf muscle and Achilles tendon properties in children with CP compared to TD. Specifically, muscle fascicles in CP children were found to be stiffer than the tendon,^22,41^ and shorter than in TD,^11,49^ limiting the muscles’ ability to passively lengthen.^7^ Furthermore, the Achilles tendon in CP was found to be longer than typical,^8,49^ possibly compensating for the shorter fascicle length. Evidence is conflicting about whether the Achilles tendon in CP is also less stiff^22,25^ or not^41^ compared with TD. While it is generally understood that musculotendon properties affect muscle efficiency and energy demands,^27,34^ we do not know exactly how these adaptations in musculotendon tissue properties affect the plantarflexors’ mechanical behaviour during functional tasks in individual patients with CP.

Neuromusculoskeletal modelling can enhance our understanding of how aberrant musculotendon properties affect functional activities, such as gait.^17,33,47^ Ultimately, such understanding will aid clinical decision making, for example to address whether or not to treat such aberrant properties. In these cases it is important that musculotendon model parameters account for a subject’s specific musculotendon mechanics. For example, subject-specific musculotendon model parameters substantially affect estimated muscle forces,^5,44^ joint contact forces,^14^ and metabolic energy consumption^4^ during gait. Yet, it remains unclear to precisely what extent default musculotendon model parameters, typically based on elderly cadavers,^3,16,46^ are representative of children, let alone, of children with CP. Earlier work has attempted to estimate musculotendon properties for muscles in CP,^17,43^ but were not informed by ultrasound data. Further insights into how to personalise plantarflexor musculotendon parameters for children with CP is necessary to apply personalised simulations to evaluate musculotendon function in individual patients.

Hence, the aims of our study were to estimate personalised musculotendon parameters that capture the mechanical behaviour of the plantarflexors and compare these personalised parameters to the model’s default parameters and to those personalised for TD children.

## Materials and Methods

**Figure 1:**
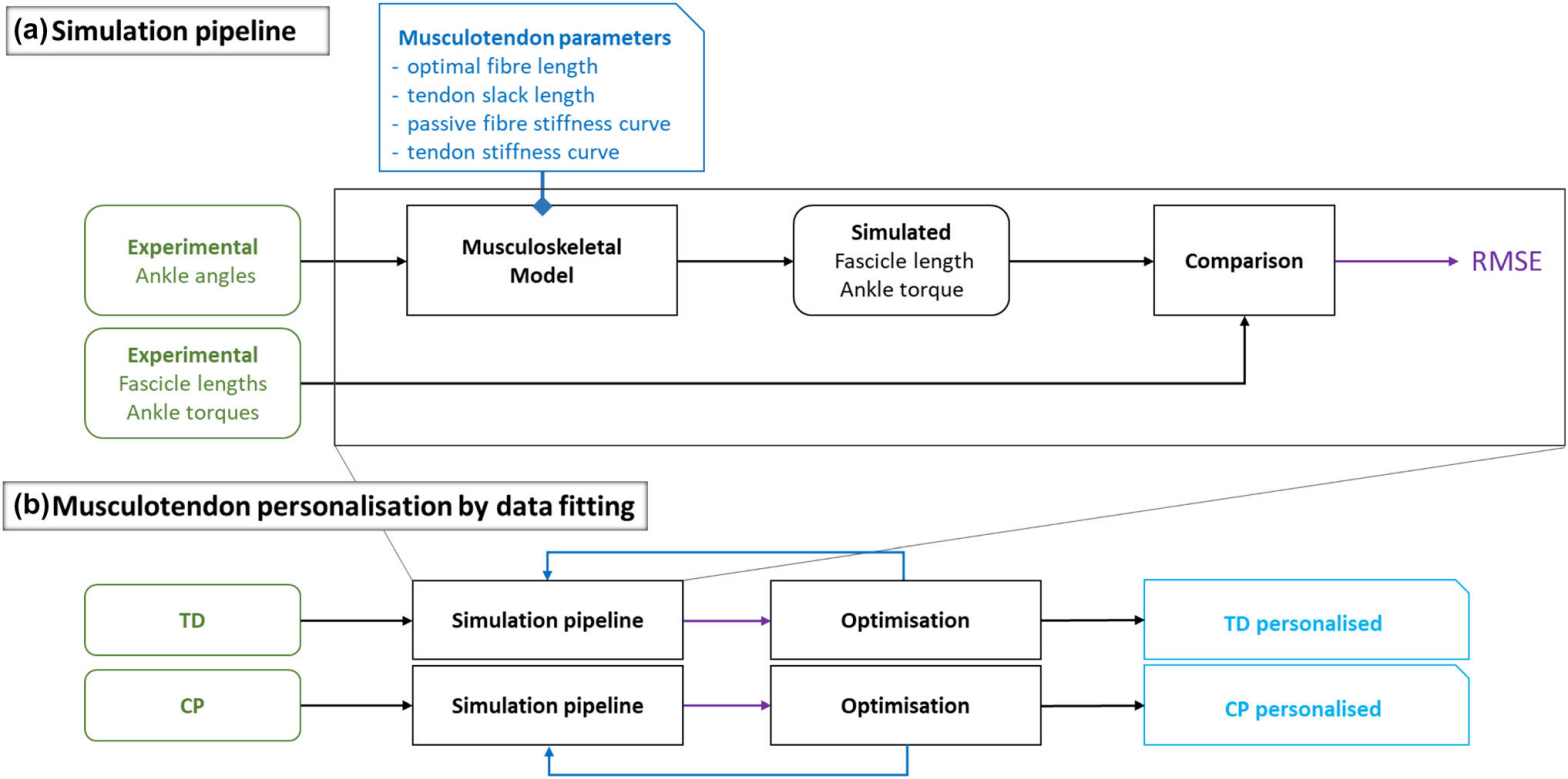
Overview of the Study Methodology. (**a**) The simulation pipeline employed to compare simulated muscle behaviour to experimental data for different musculotendon parameters that resulted in the difference (root mean square errors (RMSE)) between simulated ankle torque and fibre length and measured ankle torque (load-cell) and fascicles length (ultrasound). (**b**) The simulation pipeline was employed in the optimisation cost function to minimise the sum of squared differences (a least squares fit) between simulated and experimental quantities (from **a**) to obtain personalised musculotendon parameters for both a TD dataset and CP dataset. Personalised parameters were compared between groups and to default.

We applied musculoskeletal modelling to simulate passive ankle torque and medial gastrocnemius fascicle length during slow passive ankle rotation for 13 children with CP and 13 TD children, and compared these quantities to corresponding experimental measures by calculating root mean square errors (RMSEs). Next, we fitted musculotendon parameters to minimise these errors and yield personalised parameters for all subjects (Fig. 1).

### Experimental Data

**Figure 2:**
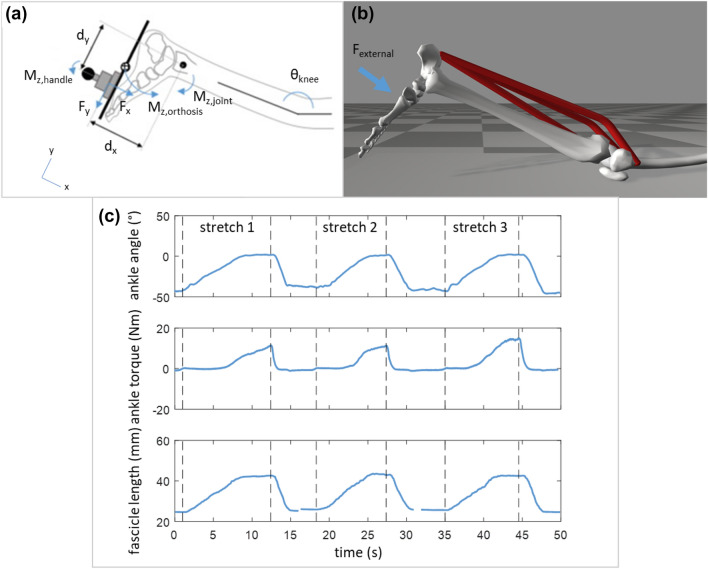
(**a**) Schematic diagram of the foot and instrumented footplate in the experimental setup. The x-axis is oriented through the middle of the handle, the *y*-axis is oriented along the footplate, and the *z*-axis orthogonally to the *x*- and *y*-axis. The sum of moments around the ankle in the sagittal plane (around the *z*-axis) is given by: ${M}_{z,\mathrm{total}}={F}_{x}{d}_{y}+{F}_{y}{d}_{x}+{M}_{z,\mathrm{handle}}+{M}_{z,\mathrm{orthosis}}-{M}_{z,\mathrm{joint}}$, where ${d}_{y}$ and ${d}_{x}$ correspond to the moment arm distances from the point of force application, respectively ${F}_{x}$ and ${F}_{y}$, of the load-cell to the lateral malleolus that is aligned with the rotation axis of the orthosis. ${M}_{z,\mathrm{handle}}$ is the moment exerted on the handle around the z direction. ${M}_{z,\mathrm{orthosis}}$ is the calculated moment caused only by the weight of the orthosis. ${M}_{z,\mathrm{joint}}$ is the applied (net muscle) moment around the ankle joint. The moment of inertia for both foot and footplate are neglected, because the mass of both is relatively small the angular acceleration is negligible. Adapted from Kalkman et al. (2017).^23^ (**b**) The OpenSim model representation of the experimental setup, which defines the three musculotendon units of the plantarfexors (GASL, GASM, and SOL) about the ankle joint that were simulated. (**c**) Example data for each of the experimentally measured variables over the time for a representative subject. Variables were normalised over each stretch cycle and averaged for each subject, to obtain torque–angle and fascicle length-angle curves.

Data of 13 children with spastic CP and 13 TD children, collected as part of a larger study,^22^ were used (Table 1). TD children were selected from a dataset of 16 children in such a way that the groups were matched in height. Age, mass and tibia length did not differ significantly between groups. The data collection protocol was approved by the National Health Service research ethics committee in the UK and the University Hospital’s ethics committee in Leuven, Belgium. Subjects lay prone on a bed with their lower leg supported at a knee flexion angle of 20° (schematically displayed in Fig. 2). The lower leg was positioned in an orthosis, consisting of a rigid footplate that was strapped to the foot and an adjustable insole to ensure heel contact with the footplate. Videos were also made to control that the heel did not lose contact with the footplate. The rotation axis of the orthosis was aligned with the lateral malleolus. The foot was manually rotated from plantarflexion to maximal dorsiflexion, with a maximal angular velocity of 15 ± 5°/s. This slow velocity was selected to not provoke a stretch reflex.^10^ The rotations were performed by the same person for all subjects. Surface electromyography (sEMG) of the lateral gastrocnemius (GASL), soleus (SOL) and tibialis anterior was collected at 1600 Hz. Marker clusters on the footplate and shank were recorded with three cameras at 120 Hz (Optitrack, Corvallis, OR, USA). Applied forces and torques around the ankle were measured at 200 Hz using a load-cell (ATI mini45; Industrial Automation, Apex, NC, USA) attached to the orthosis. A B-mode ultrasound scanner (Telemed Echoblaster, Vilnius, Lithuania) was secured over the medial gastrocnemius (GASM) mid-muscle belly to visualise fascicle length changes at 60 Hz. Leg length, tibia length and foot length were also measured per subject.^22^

**Table 1:** Participant charasteristics.

	TD (*n* = 13)	CP (*n* = 13)
Age (years)	11.1 ± 3.4	11.6 ± 3.1
Sex	6 male, 7 female	10 male, 3 female
GMFCS	n/a	8 × I, 5 × II
Mass (kg)	39.2 ± 14.4	37.5 ± 19.0
Height (cm)	143.7 ± 17.6	143.7 ± 21.4
Tibia length (cm)	33.0 ± 0.5	34.2 ± 0.6

Mean ± standard deviation are shown.

*CP* cerebral palsy; *GMFCS* gross motor functional classification system;^36^ and *TD* typically developing.

Data were processed in Matlab R2015a and Python 2.7.11. Ankle angles were obtained by the relative orientation of the anatomical reference frames^26^ of the shank and foot. The net ankle joint moment was calculated from the exerted torques and forces on the load cell and the estimated torque caused by gravity on the orthosis (Fig. 2a). Both the net ankle joint torque and the marker data were filtered with a second order lowpass 6 Hz Butterworth filter. GASM fascicle length was defined as a straight-line distance between the upper and lower aponeurosis along the lines of collagenous tissue.^22^ Both aponeuroses and the fascicle length were manually defined in the first frame of the ultrasound images, and tracked by semi-automatic tracking software^13,18^ for the rest of the video to estimate fascicle lengthening. EMG data was only used to control that the movements were performed passively. Trials were discarded when the root mean square of the processed sEMG signal exceeded 10% of the maximal voluntary contraction, as this indicated that the stretch was not performed passively.^22^ All variables (i.e., ankle angle, ankle torque, and fascicle lengthening) were calculated during three ankle stretches per subject (example in Fig. 2c). Then, the variables were time-normalised to have the same number of samples for each stretch. Subsequently, average torque–angle and fascicle length-angle curves per subject were obtained by taking the time-normalised average of the variables over the stretches. Since we were only interested in modelling plantarflexor stretch, the average curves were cropped from the point when the internal plantarflexion moment was higher than 0 Nm up to the point when the maximum dorsiflexion angle was reached (end range-of-motion).

### Generating Scaled Musculoskeletal Models

A generic full body musculoskeletal model^37^ with default parameters representative of the lower-limb musculature of unimpaired individuals was used as the starting point to model children in OpenSim 4.0.^40^ All joints were locked at 0°, except for the left knee, which was locked at 20° of flexion as imposed during the measurements, and the assessed ankle, which was free to move. All muscles were removed except for the main plantarflexors: GASL, GASM, and SOL (Fig. 2b). The model’s lower-limb dimensions were linearly scaled to individual subject measurements of foot, tibia and leg length. Along with the body segment sizes, the muscles’ default optimal fibre length and tendon slack length were scaled by OpenSim to maintain the original optimal fibre length to tendon slack length ratio. The maximal isometric muscle forces (${F}^{\mathrm{max}}$) were scaled according to the ratio of total body mass ($m$):^43^

$${F}_{\mathrm{subject}}^{\mathrm{max}}={F}_{\mathrm{generic}}^{\mathrm{max}}{({m}_{\mathrm{subject}}/{m}_{\mathrm{generic}})}^\frac{2}{3}$$

This way, the model with default parameters was created for each subject.

**Figure 3:**
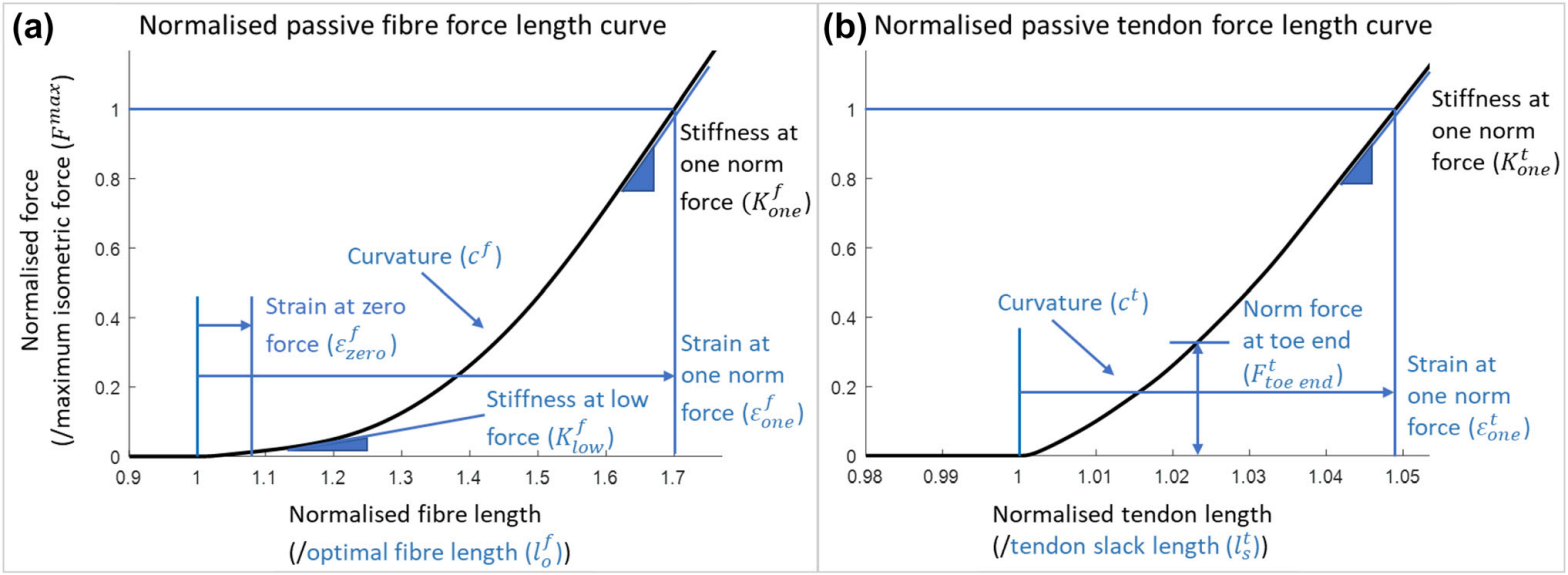
The default (**a**) normalised passive muscle fibre force–length curve and (**b**) normalised tendon force–length curve, as implemented as a Millard2012 musculotendon unit in OpenSim. All parameters defining the curves are shown, with independent parameters optimised in this study indicated in blue.

Each musculotendon unit was modelled as a Hill-type muscle model.^29^ The passive force–length curves for the muscle fibre and tendon were described by monotonically increasing functions defined by 11 parameters – 6 for the fibre and 5 for the tendon (Table 2; Fig. 3). The number of independent parameters was 9, as stiffness parameters were related to the strain parameters according to Millard et al.^29^ Length scaling factors and passive force–length curve properties were the same for all three musculotendon units, since the experimental method did not enable us to differentiate between muscle units contributing to the torque, while fascicle lengthening was only measured for the GASM.

**Table 2:** Parameters defining the passive muscle fibre- and tendon force–length curves in the Millard 2012 musculotendon unit in OpenSim.

Parameter name	Abbrev	Description	Parameter definitions ($x$ is optimised)	OpenSim default of $x$	Min of $x$	Max of $x$
Passive fibre force						
Optimal fibre length	${l}_{o}^{f}$	Optimal length of the muscle fibres, dependent on subject size	$x$*${l}_{o}^{f}$	1	0.5	2
Strain at zero force	${\varepsilon }_{\mathrm{zero}}^{f}$	Fibre length difference with ${l}_{o}^{f}$, normalised by ${l}_{o}^{f}$, at which passive force starts engaging	$x$	0	-0.5	0.5
Strain at one norm force	${\varepsilon }_{\mathrm{one}}^{f}$	Fibre length difference with ${l}_{o}^{f}$, normalised by ${l}_{o}^{f}$, at which the fibre develops ${F}^{\mathrm{max}}$	$x$	0.70	0.5	1.5
Stiffness at low force scaling factor	${K}_{\mathrm{low}}^{f}$	The normalised stiffness (i.e., slope of the force–length curve) when the fibre is just starting to develop tensile force	$x$/(${\varepsilon }_{\mathrm{one}}^{f}$-${\varepsilon }_{\mathrm{zero}}^{f}$)	0.14	0.01	0.99
Stiffness at one norm force^a^	${K}_{\mathrm{one}}^{f}$	The normalised stiffness (i.e., slope of the force–length curve) when the fibre develops ${F}^{\mathrm{max}}$	2/(${\varepsilon }_{\mathrm{one}}^{f}$-${\varepsilon }_{\mathrm{zero}}^{f}$)	n.a	n.a	n.a
Curvature	${c}^{f}$	The curvature of the force–length relationship between stiffness at low force to stiffness at one norm force	$x$	0.75	0	1
Tendon force						
Tendon slack length	${l}_{s}^{t}$	Resting length of the tendon, dependent on subject size	$x$*${l}_{s}^{t}$	1	0.5	2
Strain at one norm force	${\varepsilon }_{\mathrm{one}}^{t}$	Tendon length difference with ${l}_{s}^{t}$, normalised by ${l}_{s}^{t}$, at which the tendon develops ${F}^{\mathrm{max}}$, determining tendon compliance	$x$	0.049	0.01	5
Stiffness at one norm force^a^	${K}_{\mathrm{one}}^{t}$	The normalised stiffness (i.e., slope of the force–length curve) when the tendon develops ${F}^{\mathrm{max}}$	1.375/${\varepsilon }_{\mathrm{one}}^{t}$	n.a	n.a	n.a
Norm force at toe end	${F}_{\mathrm{toe end}}^{t}$	The normalised force developed at the end of the ‘toe’ region, which lies between 0 strain and some intermediate strain less than the strain required to develop ${F}^{\mathrm{max}}$	$x$	0.67	0.01	0.99
Curvature	${c}^{t}$	The curvature of toe region of the force–length relationship	$x$	0.5	0	1

The fourth column indicates parameters $\mathrm{x}$ that were optimised within constrained values (sixth and seventh column) that were set based on model constraints and to keep parameters within reasonable bounds.

^a^Parameters that were completely dependent on other parameters for simplicity, and therefore not considered for optimisation; ${\mathrm{F}}^{\mathrm{max}}$ is the maximum isometric muscle force.

### Simulating Ankle Torque and Muscle Fascicle Length

Muscle-generated ankle torque and GASM fascicle length were simulated by solving for muscle fibre and tendon equilibrium at each ankle angle in OpenSim 4.0. Muscle activations were set to 0 to represent a passive stretch. Net ankle torque and GASM fascicle length were computed per subject using their scaled musculoskeletal model, with their measured ankle angle over time as input. The calculated individual muscle moments were summed to obtain the net plantarflexor torque over time. The ankle torque and GASM fascicle length were interpolated over ankle angle to get the ankle torque–angle and fascicle length-angle curve per subject.

### Personalising Muscle Model Parameters

The passive musculotendon model parameters (i.e., the 9 independent parameters in Table 2) were personalised by fitting the model to experimentally measured ankle torque–angle and GASM fascicle length-angle curves. A nonlinear curve-fitting least-squares solver (*lsqcurvefit;* Matlab 2016a, The Mathworks) was used to minimise the error between simulated and measured ankle torque and GASM fascicle length over the ankle angle. To maintain similar relative weightings and thus an equal match between torque and length curve-fitting, fascicle lengths were expressed in millimetres to be in the same order of magnitude as the torque data in Nm. For the muscle model parameters to be optimised (Table 2), their default values were used as the initial guess (Table 2, column 5). They were constrained within bounds (± , Table 2, columns 6 and 7) to ensure that the optimised parameters remained within a reasonable range and adhered to model constraints that were defined by the Millard muscle model. As ${l}_{o}^{f}$ and ${l}_{s}^{t}$ depend on subject size, their scaling factors, rather than absolute values, were optimised, allowing for between-subject comparisons. RMSEs were calculated between measured and simulated torque–angle and fascicle length-angle curves. Ankle torque was normalised to bodyweight and fascicle length was normalised to tibia length to facilitate comparisons between participants.

The following stepwise approach was used to evaluate which (combination of) fitted parameters best reduced the RMSEs. First, RMSEs were calculated using the default parameters. Second, each parameter was optimised independently, and the parameter that reduced median RMSEs the most was selected to be included into the personalisation. Second, each remaining parameter was fitted in combination with the first parameter, and, again, the parameter that further reduced the RMSEs the most was included in the optimisation. Iterations continued until the fit RMSEs were overall below 10% of their default values. Additionally, the median values from the personalised parameters, considered to be group-specific parameters for TD and CP separately, were applied and RMSEs were calculated to evaluate the effects of using group-specific medians. The Matlab code to create a model with group-specific median parameters is freely available at https://simtk.org/projects/median_pfmodel.

The personalised parameters for CP and TD were compared to default values by One-Sample Wilcoxon Signed Rank Test, and between TD and CP using Man-Whitney *U* tests, since all outcomes were non-normally distributed as shown by Kolmogorov–Smirnov tests. Also, default and group-specific median RMSEs were compared by Man-Whitney U tests. Matlab (2016a) was used for all statistical analyses. Significance level was set at $p<0.05$.

## Results

### Musculotendon Parameter Personalisation

**Figure 4:**
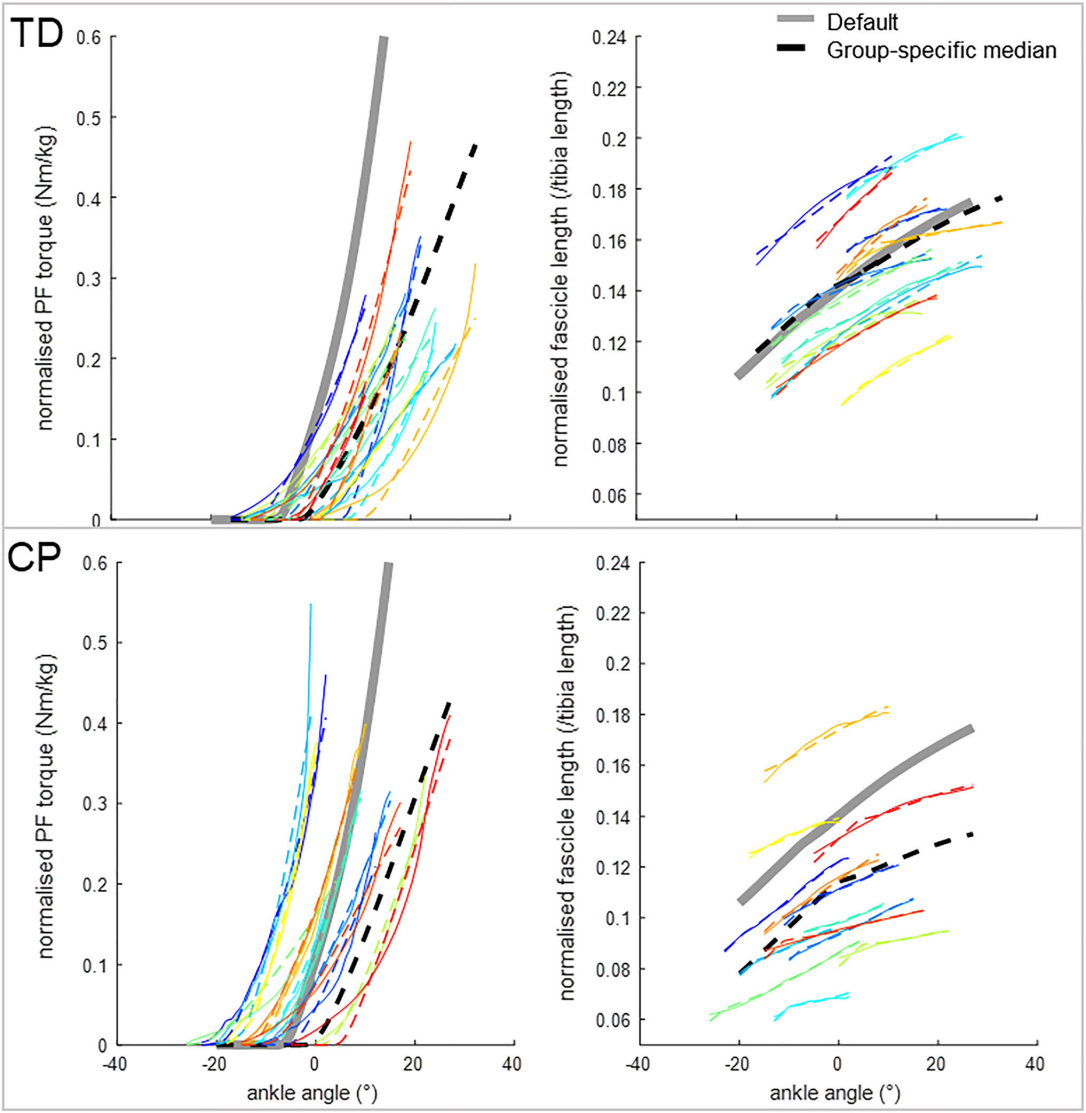
Individual measured (solid coloured) and optimised (dashed coloured lines), model default (grey lines), and model with group-specific median parameters (dashed black line) plantarflexor (PF) torque–angle (left) and fascicle length-angle (right) curves for TD and CP. Each colour indicates a different individual.

**Figure 5:**
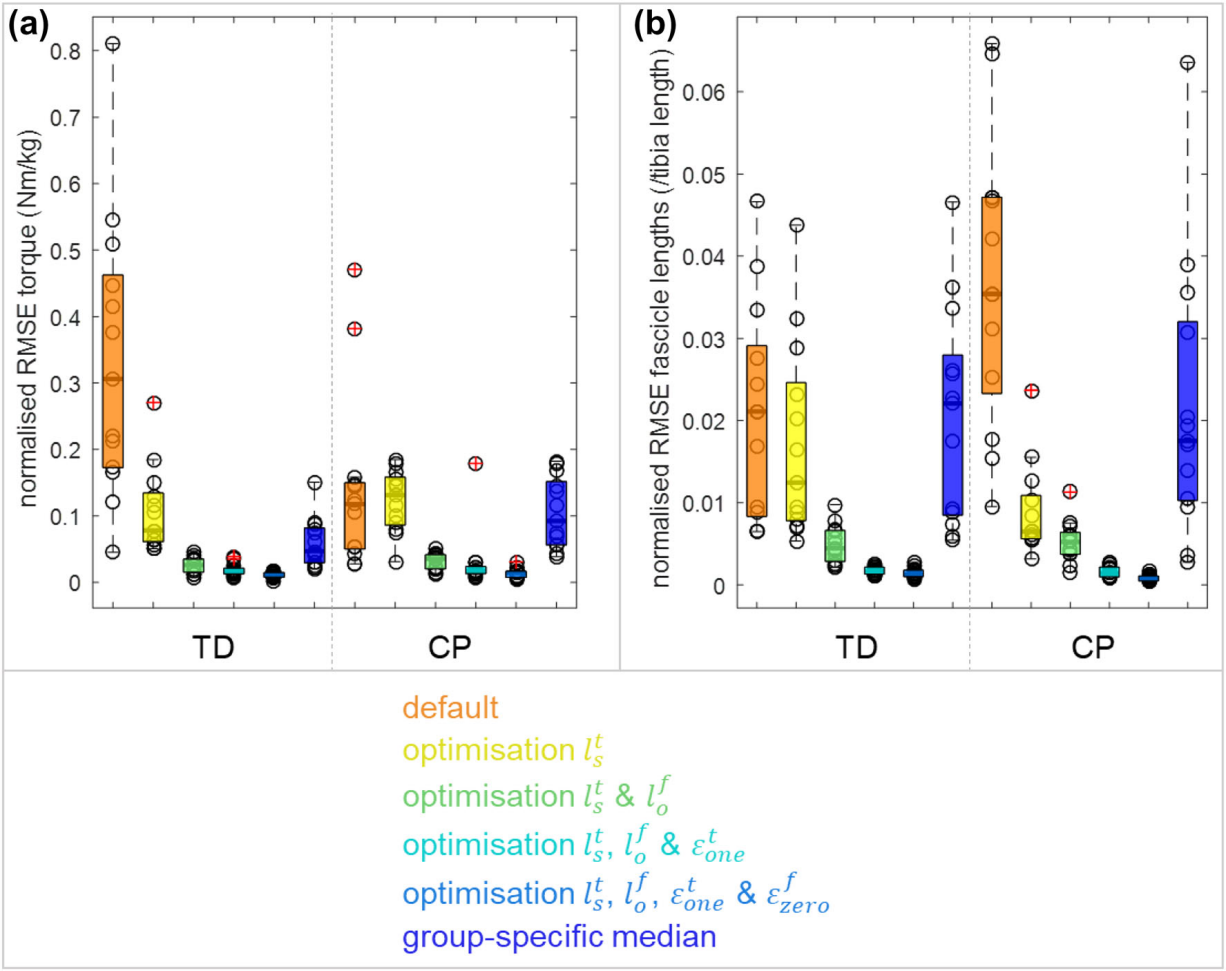
The RMSE with experimental data for (**a**) the torque–angle and (**b**) fascicle length-angle curves for TD children and children with CP for default parameters, in each step for the best fit parameters, which include tendon slack length (${l}_{s}^{t}$), optimal fibre length (${l}_{o}^{f}$), tendon strain at one norm force (${\upvarepsilon }_{\mathrm{one}}^{t}$), and fibre strain at zero force (${\upvarepsilon }_{\mathrm{zero}}^{f}$), and for group-specific median parameters. Circles indicate individual values. Boxplots display median values and interquartile ranges, red ‘ + ’ indicate outliers.

With default musculotendon parameters, the overall fit between simulated and experimental torque–angle and fascicle length-angle curves was not good (Fig. 4, thick grey line versus solid colored lines). The RMSE for torque was particularly poor for TD (0.12 [0.10] in CP vs. 0.31 [0.29] Nm/kg in TD (median [IQR])), while RMSE for fascicle length was particularly poor for CP (0.035 [0.024] in CP vs 0.021 [0.021]/tibia length in TD) (Fig. 5).

The most important parameters to personalise were, in stepwise order, ${l}_{s}^{t}$, ${l}_{o}^{f}$, ${\varepsilon }_{\mathrm{one}}^{t}$, and ${\varepsilon }_{\mathrm{zero}}^{f}$ (see Online Appendix A for results of each step). Adding a fifth parameter resulted in median RMSEs below 10% of default RMSEs, as well as parameter redundancy, as shown by fibre parameters reaching extreme values for some subjects. By adding each personalised parameter, RMSE reduced for both torque and fascicle length. When fitting all four parameters, RMSE for normalised torques was 0.014 [0.0091] for CP and 0.012 [0.0066] Nm/kg for TD, and RMSE for normalised fascicle length was 0.00076 [0.00059] for CP and 0.0012 [0.00076] /tibia length for TD. Hence, by fitting, RMSE was reduced by 88% for torque and by 98% for fascicle length compared to default for CP. For TD, torque RMSE was reduced by 96% and fascicle length by 94%.

When applying the group-specific median parameter values (Table 3), normalised torque RMSE was 0.092 [0.095] for CP and 0.047 [0.052] Nm/kg for TD. Fascicle length RMSE was 0.018 [0.022] for CP, and 0.022 [0.020] /tibia length for TD. Differences compared to default RMSE were not significant for torque in CP (*p* = 0.96) and for fascicle length in TD (*p* = 1.0). But, with group-specific medians, RMSE was reduced significantly by 51% for fascicle length (*p* = 0.027) compared to default for CP, and torque RMSE was reduced by 84% (*p* < 0.001) for TD.

**Table 3:** Overview of the parameter values in the personalised models.

Parameter name	Abbrev	Personalised parameters				Default value
		CP		TD		
		Median	IQR	Median	IQR	
Passive fibre force						
Optimal fibre length Scaling factor Normalised value (/tibia length) GASM GASL SOL	${l}_{o}^{f}$	0.81^#~^ 0.10 0.12 0.09	0.27 0.04 0.04 0.03	1.15^#^ 0.15 0.17 0.13	0.23 0.03 0.03 0.02	1.00 0.13 0.15 0.11
Strain at zero force	${\varepsilon }_{zero}^{f}$	0.089	0.30	-0.055	0.25	0.00
Stiffness at low force scaling factor*	${K}_{low}^{f}$	0.23	0.09	0.19	0.07	0.14
Stiffness at one norm force*	${K}_{one}^{f}$	3.28	1.27	2.65	0.94	2.86
Tendon force						
Tendon slack length Scaling factor Normalised value (/tibia length) GASM GASL SOL	${l}_{s}^{t}$	1.03^#~^ 1.04 0.98 0.72	0.03 0.01 0.01 0.01	1.00 1.00 0.95 0.70	0.03 0.03 0.03 0.03	1.00 1.00 0.95 0.70
Strain at one norm force	${\varepsilon }_{one}^{t}$	0.23^#^	0.14	0.16^#^	0.14	0.049
Stiffness at one norm force*	${K}_{one}^{t}$	6.06	4.69	8.47	5.95	28.1

Parameters that were included in the optimisation to be personalised are indicated in blue. Parameters that were dependent on the personalised parameters are indicated by an asterix (*). Optimal fibre length and tendon slack length for each muscle value were normalised to tibia length (Table 1) since depending on subject size.

^#^Indicates that personalised values are significantly different from default, and ^~^ indicates that CP values are significantly different from TD.

**Figure 6:**
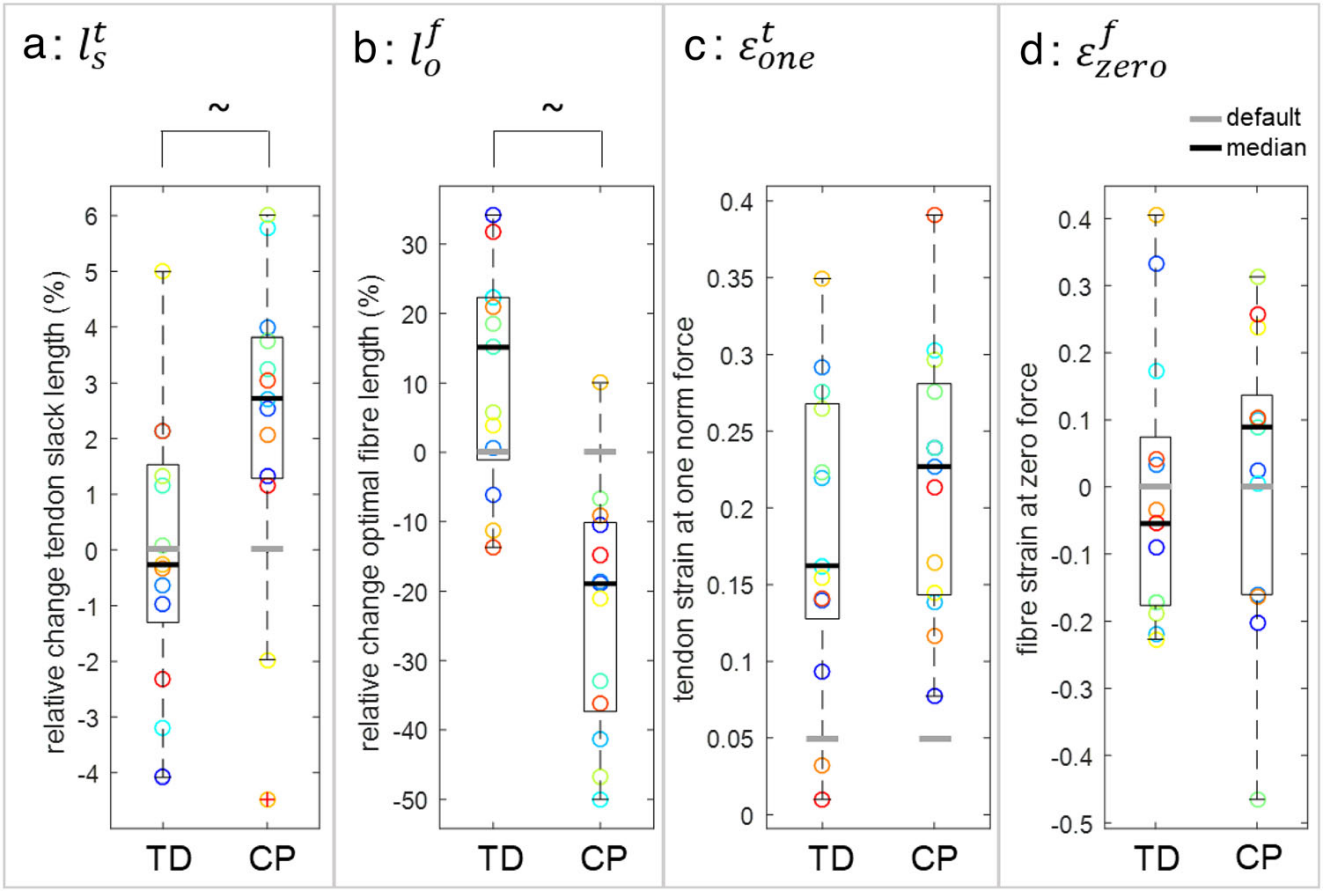
Personalised (**a**) relative change of ${l}_{s}^{t}$ from the default value, (**b**) relative change of ${\mathrm{l}}_{\mathrm{o}}^{\mathrm{f}}$ from the default value, (**c**) ${\upvarepsilon }_{\mathrm{one}}^{\mathrm{t}}$, and (**d**) ${\upvarepsilon }_{\mathrm{zero}}^{\mathrm{f}}$ for CP and TD. Coloured circles indicate individual values, matching colours with the individual curves in Fig. 5. Boxplots display median values (thick black line) and interquartile ranges, red ‘ + ’ indicate outliers. The thick grey lines indicate default values. ~ indicates significant differences between CP and TD.

When comparing the personalised musculotendon parameters from CP to default values and to TD (Table 3; Fig. 6), in CP, ${l}_{s}^{t}$ were longer than default (*p* = 0.03) and than in TD (relative change from default CP: + 2.7% vs. TD: + 0.3%, *p* = 0.02). Personalised TD ${l}_{s}^{t}$ did not differ significantly from default (*p* = 0.95). Personalised CP ${l}_{o}^{f}$ were shorter than default (*p* = 0.001) and than TD (relative change from default CP: − 19% vs. TD: + 15%; *p* < 0.001). Personalised TD ${l}_{o}^{f}$ were longer than default (*p* = 0.001). ${\varepsilon }_{\mathrm{one}}^{t}$ was higher than default for both groups (*p* < 0.001 for CP, *p* = 0.001 for TD), and did not differ significantly between CP and TD (0.23 in CP vs. 0.16 in TD, *p* = 0.38). ${\varepsilon }_{\mathrm{zero}}^{f}$ did not differ significantly from default for CP (*p* = 0.59) and TD (*p* = 0.59), and personalised values did not differ significantly between groups (0.089 for CP vs. − 0.055 for TD, *p* = 0.41).

## Discussion

We aimed to estimate personalised musculotendon parameters that capture the mechanical behaviour of the plantarflexors during a slow passive ankle stretch for children with CP, and compare these personalised parameters to default parameters and to those personalised for TD children. We determined that default OpenSim musculotendon parameters based on a default, adult model^37^ resulted in relatively poor fits for ankle torque for TD children and for fascicle length for children with CP. Expectedly, fits improved when personalising parameters based on experimental data, by more than 88% for torque, and more than 94% for fascicle length. Personalised parameters reflected CP-specific musculotendon adaptations, with tendon slack length (${l}_{s}^{t}$) being longer and optimal fibre length (${l}_{o}^{f}$) being shorter than default and compared to TD children. In addition, personalised tendon compliance (tendon strain at one norm force;${\varepsilon }_{\mathrm{one}}^{t}$) was significantly increased in both groups compared to default. Using group-specific median parameters significantly improved the torque match by 84% in TD, and the fascicle length match by 51% in CP compared to default parameters. These results indicate that default parameters are not representative for children with CP and that personalisation of parameters by fitting to experimental data can be achieved to create a model that describes passive plantarflexor mechanical behaviour more accurately. Using the CP-specific median values results in accuracy improvements for fascicle lengths compared to default, but is inadequate for capturing the variability in the experimental behaviour in CP.

Comparing the default model fit with experimental data revealed that, on average, the default model is too stiff for TD children, whilst showing a better fit for torque of CP than of TD. Personalised optimal fibre length was significantly longer than default in TD. Hence, this suggests that scaled default model parameters that are based on healthy older adults, are not directly representative for TD children, which is in agreement with previous findings for the hamstrings.^43^ This premise is also supported by experimental findings of patellar^32^ and Achilles^48^ tendons showing greater strains for equal stresses in children than in adults due to a lower elastic modulus. Indeed, through data fitting, it was found that the default tendon compliance scaled to height and maximum muscle force was on average too low for both groups. In earlier studies evaluating healthy adult gait, Achilles tendon compliance was already increased (from 0.049 to 0.10) to be more consistent with experimental data and to account for the plantarflexor aponeurosis in the model,^2,33,45^ but our results indicate that its value should be increased even further for children, to 0.16 for TD and 0.23 for CP on average. Alternatively, it has also been argued that even for healthy adults, modelled passive muscle stiffness is too high, for example for the vasti,^3^ indicating inaccuracies in describing the muscles’ mechanical behaviour by the default model. The default fascicle length fitted relatively well for TD children, but was too long for children with CP, agreeing with experimental findings of shorter fascicles in CP compared to TD.^8,11,30,49^ This was reflected by personalised longer tendons and shorter fascicles in CP compared to TD after data fitting in height-matched groups. Even though the fitted parameters could not directly be validated, we found 4% higher normalised tendon slack length in CP than in TD, which is comparable to an experimentally reported value of 6%.^41^ Optimal fibre length was 30% shorter in CP than in TD in our study. Previously, a significant reduction of normalised fascicle length of 10% has been reported for the medial gastrocnemius between CP and TD when measured at resting length.^30^ This difference is smaller than found in our study, but the comparison is not completely fair. Fascicle length values depend on the ankle angle at which they are measured, and it is unclear at what range of the force–length curve the muscle was operating experimentally. It has been reported that in CP, fascicles operate at longer than normal sarcomere lengths,^20,28^ which may also explain the difference. A direct comparison of optimal fibre lengths cannot be made, as it has not been measured *in vivo* in children. Since none of the other personalised parameters differed significantly between CP and TD, our findings indicate that the higher muscle and lower tendon stiffness in CP than in TD as measured previously^22,41^ are fully captured in the model by optimal fibre length and tendon slack length adaptations.

Personalised musculotendon parameters obtained through data fitting captured the passive plantarflexor mechanical behaviour accurately. The best fit RMSEs were within previously reported standard error of measurement (SEM) values for the torque (RMSE: 0.49 Nm; SEM: 0.72 Nm^9^) and fascicle length (RMSE: 0.3 mm; SEM: 2.0mm^22^). SEM values for the torque were determined at a neutral ankle angle (0°), and SEM values for the fascicle length at a common range of motion (− 25 to − 5° dorsiflexion), both during slow ankle stretches. Although assessing the effects of the parameter personalisation on simulation of functional activities such as gait was beyond the aims of the current study, it is likely that outcomes will be improved, for instance in terms of muscle power and efficiency.^27^ Previous literature has shown that simulated muscle forces are sensitive to tendon slack length and stiffness parameter values,^12,27,35,38^ which were both identified as important parameters for personalisation. Moreover, previous studies have shown that parameter personalisation affected outcomes such as muscle forces,^5,44^ joint contact forces,^14^ and metabolic energy consumption^4^ during gait, further indicating the importance of personalised musculotendon parameters.

Our stepwise approach to identify parameters to be optimised (Online Appendix A) provided insights into which parameters were most important to personalise. Optimal fibre length and tendon slack length were found to be important to match the amplitude of the torque and fascicle length curves, while the strain parameters contributed to a better fit of the shape and slope of the curves. The stepwise approach also revealed the parameters’ independent effects on torque and fascicle length fitting, in which fibre parameters mostly improved torque fitting, and tendon parameters mostly affected fascicle length fitting. Leaving out each of the final four parameters from the optimisation worsened the data fitting for torque and/or fascicle length considerably (Online Appendix B), suggesting that the final personalised parameters were not redundant. As final RMSEs were within SEM, it could be argued that we overfitted the experimental data. This would not be the case when only fitting tendon slack length and optimal fibre length $({l}_{s}^{t}$ and ${l}_{o}^{f}$). However, not adding the last two parameters to the optimisation (i.e., tendon compliance (${\varepsilon }_{\mathrm{one}}^{t}$) and normalised fibre length difference from ${l}_{o}^{f}$ at which passive forces engages (${\varepsilon }_{\mathrm{zero}}^{f}$)) would result in a systematic error in shape, and not magnitude, between simulated and experimental curves (Online Appendix A). Tuning tendon compliance was mostly important for the slope of the fascicle-angle curve, and fibre length at which passive force engages for the shape of the torque–angle curve, hence, both were considered to add a relevant improvement in fitting experimental data. Therefore, we considered it unlikely that choosing these four parameters to be optimised resulted in overfitting the data.

A limitation of this study is that we assumed that the GASM fascicle lengthening is representative of the fascicle lengthening in GASL and SOL. It would be interesting as a next step to differentiate between fascicle behaviour of these three muscles by applying ultrasound to all three, to optimise muscles individually. However, even though the current personalised parameters may not be as accurate for GASL and SOL, we assume that overall the parameter values are altered more accurately compared to default. Another limitation is that we did not adjust plantarflexor geometry. It has been suggested that children with CP have changes in Achilles tendon moment arm, however, there is no consensus in which direction.^1,23^ Also, a limitation is that a purely passive dataset was used to obtain parameter values that also influence active muscle force production, such as maximal isometric force and optimal fibre length, while the found parameters could not be directly validated. Even though the scaling of maximal isometric force based on body mass is a commonly applied method, also in CP,^21,43,44^ the ratio of muscle mass to body mass could be different between CP and TD. Weakness in CP has partly been accounted for by the slightly lower body mass in our CP cohort, but calculated maximal isometric force may still be inaccurate, so future studies should evaluate other methods such as muscle volume or limb circumference. Optimal fibre length was also adjusted in our study, but, as it links the passive and active fibre force–length curves in the muscle model, it also affects active force production. Including active tests to the experimental data collection would help validate the obtained values of optimal fibre length and would allow evaluation of whether the modelled linkage between passive and active force–length curves is accurate in TD and CP. However, collecting such data comes with experimental difficulties. Due to impaired selective muscle control in children with CP,^19^ achieving voluntary maximal muscle activation is challenging and may introduce more inaccuracies in the collected data. Hence, given the marked limitations in passive ankle motion in children with CP, we consider the use of passive tests only a more feasible first step for parameter personalisation. We presented a method of personalising musculotendon model parameters with promising improvements in capturing passive musculotendon mechanics. Future studies should evaluate and compare personalised modelled musculotendon mechanics to measured behaviours during functional activities, such as gait, in which active control also plays a role. This is necessary to gain confidence that personalising mechanical parameters also results in more accurate estimates of musculotendon function. Also, our personalisation method should be compared against other musculotendon personalisation methods, such as EMG-based^14,17,44^ approaches, and hybrid EMG- and ultrasound-based approaches^15^ that personalised parameters during dynamic active tasks. Such comparisons will help test parameter robustness and could help to simplify personalisation methods, which is required as current personalisation methods are complex, and time- and cost-intensive. Even though the results with group-specific parameters were promising, their effects should be evaluated in an independent dataset for proper validation. Moreover, given the variation between subjects that cannot be accounted for with group-specific median parameters, especially in CP, future work should focus on identifying simple measures to create subject-specific models. While it has yet to be determined what level of parameter estimation accuracy is necessary for clinical applications, subject-specific models could enable more in-depth evaluation of musculotendon function and joint loading in a child with CP than using typical clinical gait analysis. For example, these evaluations have the potential to be used for rehabilitation planning, patient classification, studying disease aethiology and treatment optimisation.^24^ However, for routine use in clinical practice, further simplifications of subject-specific neuromusculoskeletal modelling and workflow execution are required, as well as extensive sensitivity analyses.^24,31,42^

We have shown that the default values of musculotendon parameters do not capture the passive musculotendon behaviour in TD children and children with CP. Accurate estimations of passive torque–angle and fascicle length-angle curves can be achieved by personalising parameters via data fitting, thereby capturing CP-specific musculotendon adaptations in tendon slack length and optimal fibre length. Our parameter personalisation approach could yield more accurate simulations of clinically relevant outcomes during functional tasks than with default parameter values, to better understand the effects of altered musculotendon properties in CP. Further work should focus on simplification of parameter personalisation, to make a step towards subject-specific simulations being applicable in the clinic.

## Supplementary Information

Below is the link to the electronic supplementary material.

Supplementary file1 (PDF 1684 kb)
