# Supplementary material for: Personalisation of Plantarflexor Musculotendon Model Parameters in Children with Cerebral Palsy
Source: Ann Biomed Eng. 2022 Nov 15;51(5):938–50. doi: 10.1007/s10439-022-03107-8 (PMC10122634; doi:10.1007/s10439-022-03107-8)

Appendix A: Stepwise analysis parameters

In this analysis it was determined which parameters were best to include into the optimisation in order to reduce RMSE with experimental torque and fascicle lengths. Parameters that were selected to be included into the optimisation in each step are indicated by *.

Step 1: Each parameter fitted independently

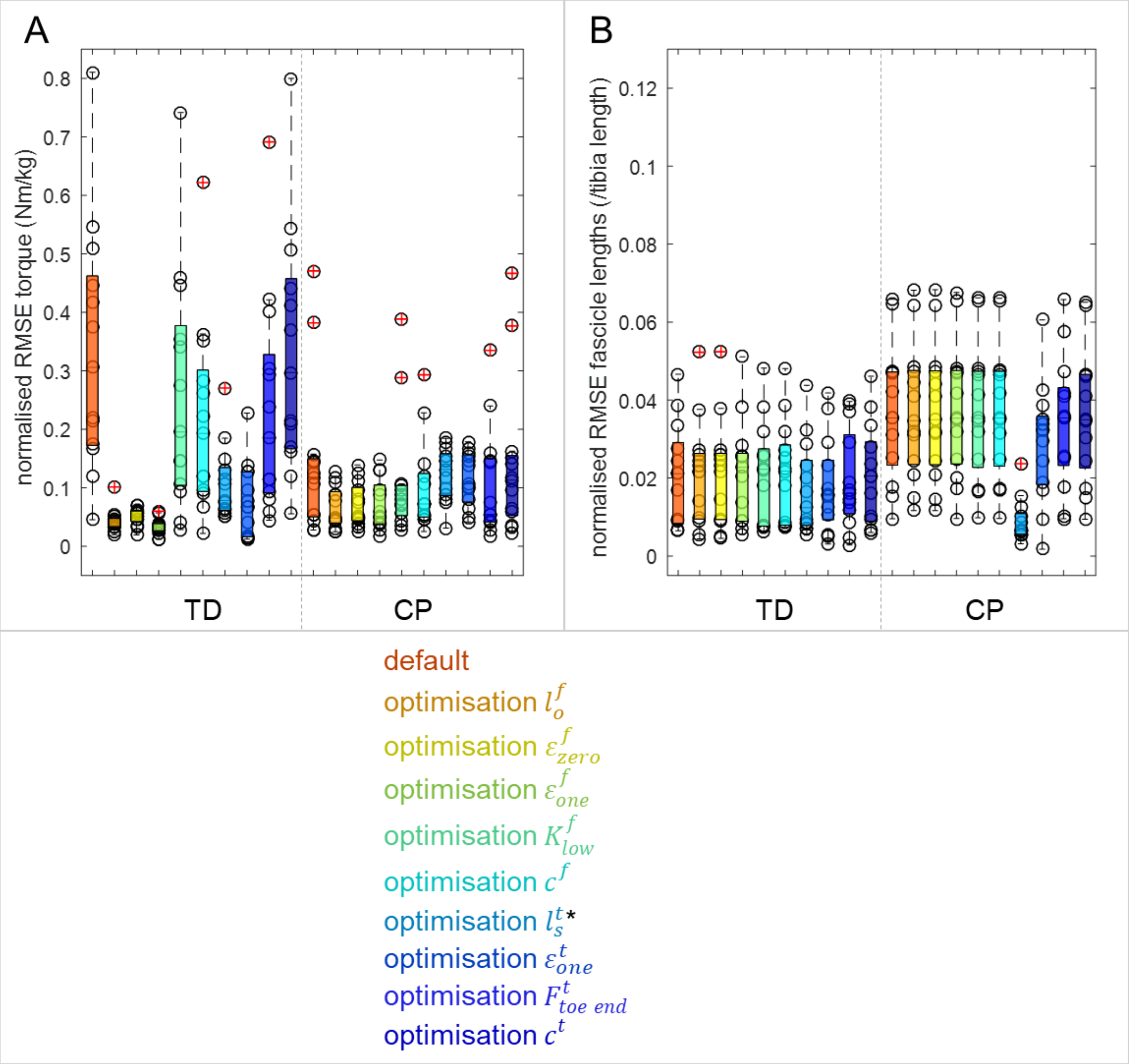

Optimisation l_s^t

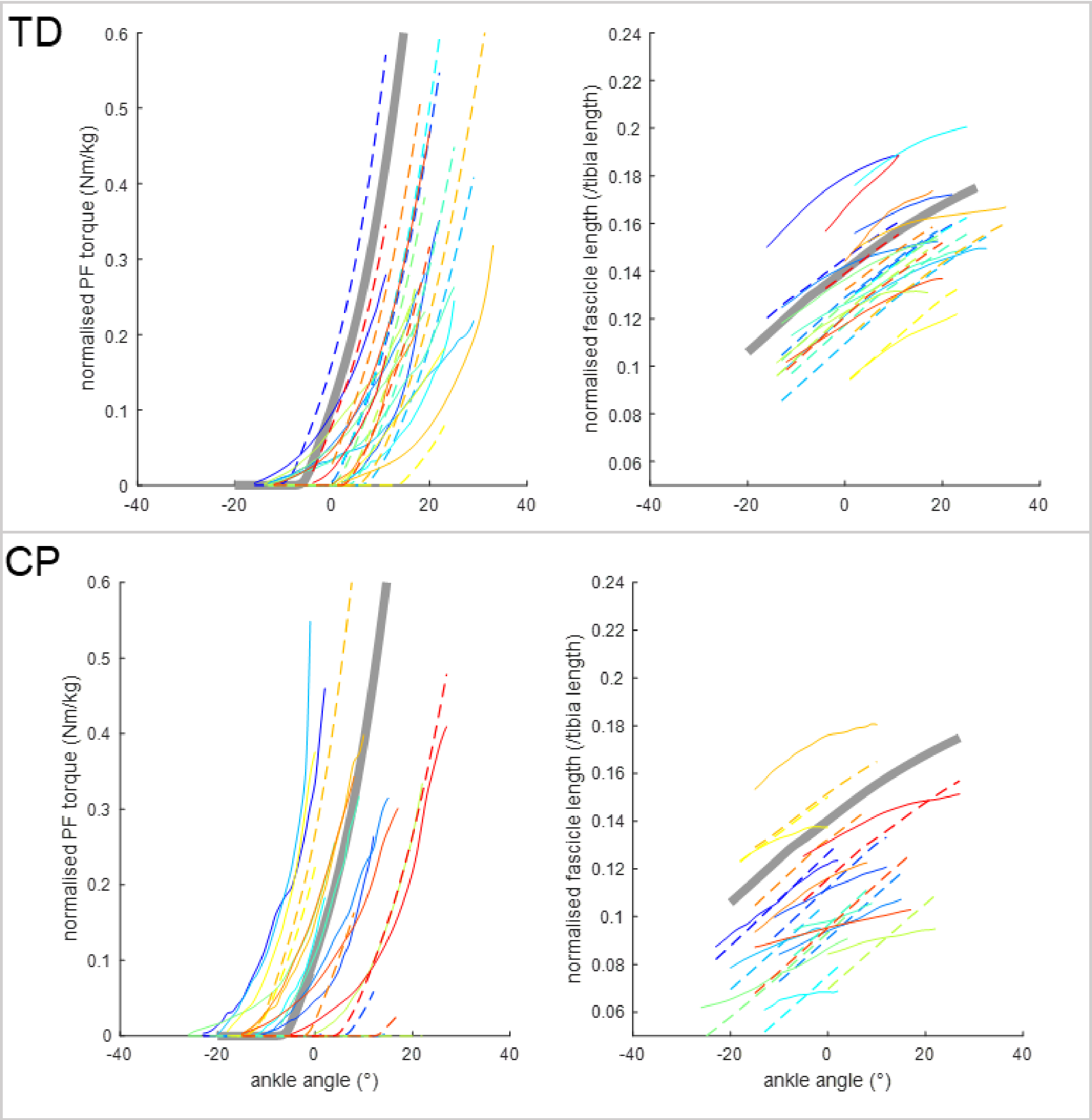

Individual measured (solid coloured) and optimised (l_s^t; dashed coloured lines), and default (grey lines) plantarflexor (PF) torque-angle (left) and fascicle length-angle (right) curves for TD and CP. Each colour indicates a different individual.

Step 2: l_s^t and each parameter

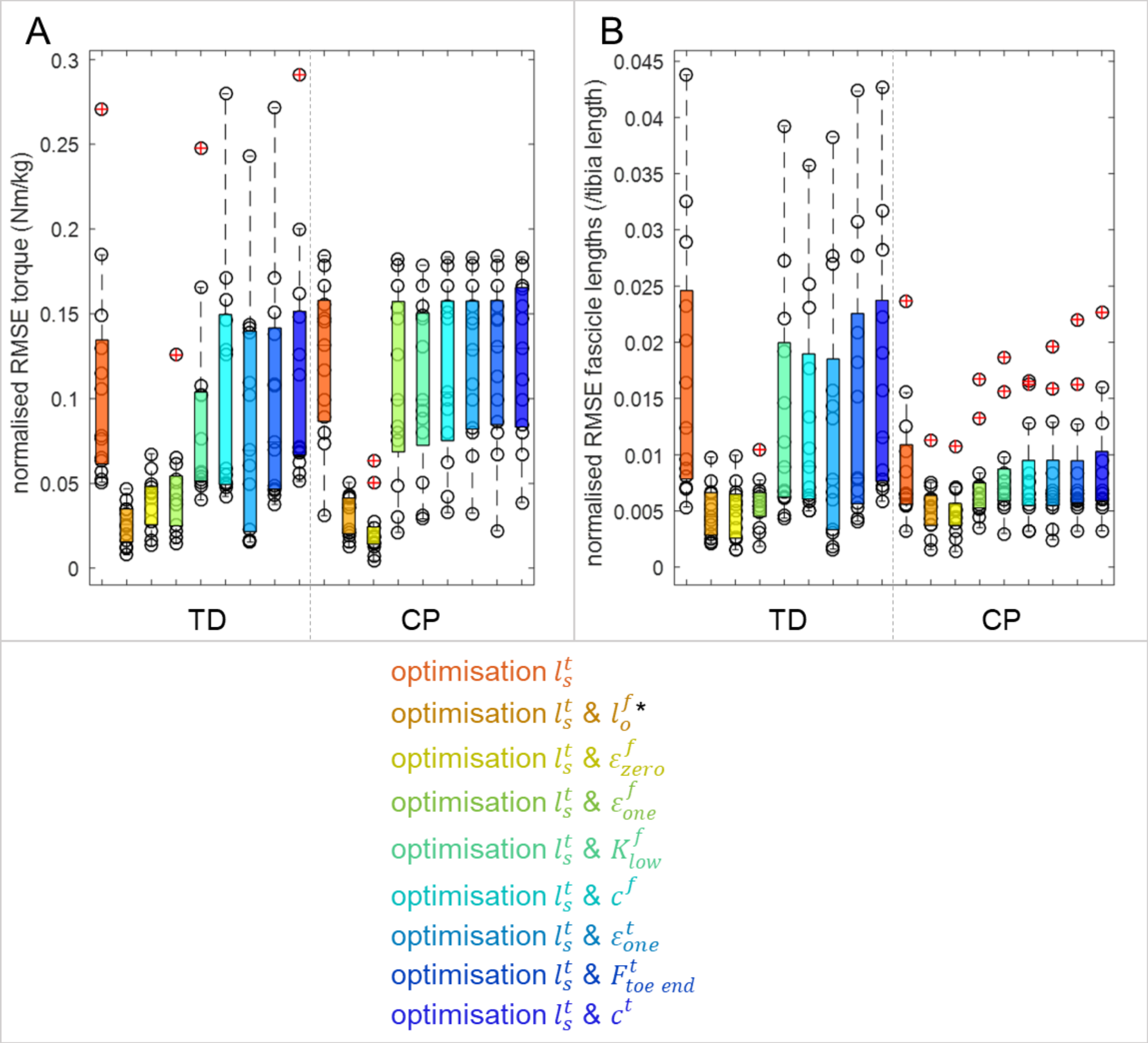

Optimisation l_s^t & l_o^f

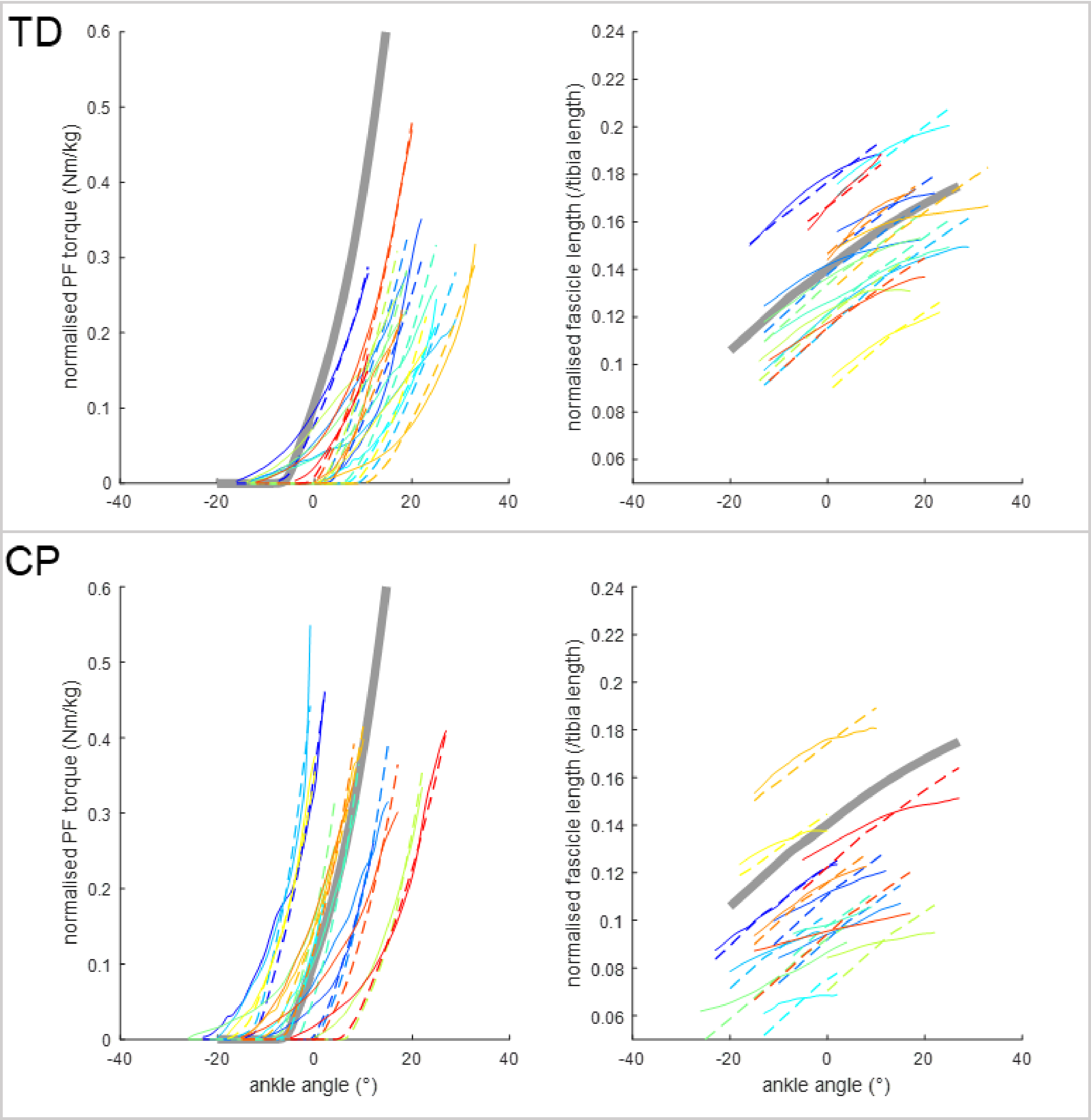

Individual measured (solid coloured) and optimised (l_s^t & l_o^f dashed coloured lines), and default (grey lines) plantarflexor (PF) torque-angle (left) and fascicle length-angle (right) curves for TD and CP. Each colour indicates a different individual.

Step 3: l_s^t, l_o^f and each parameter

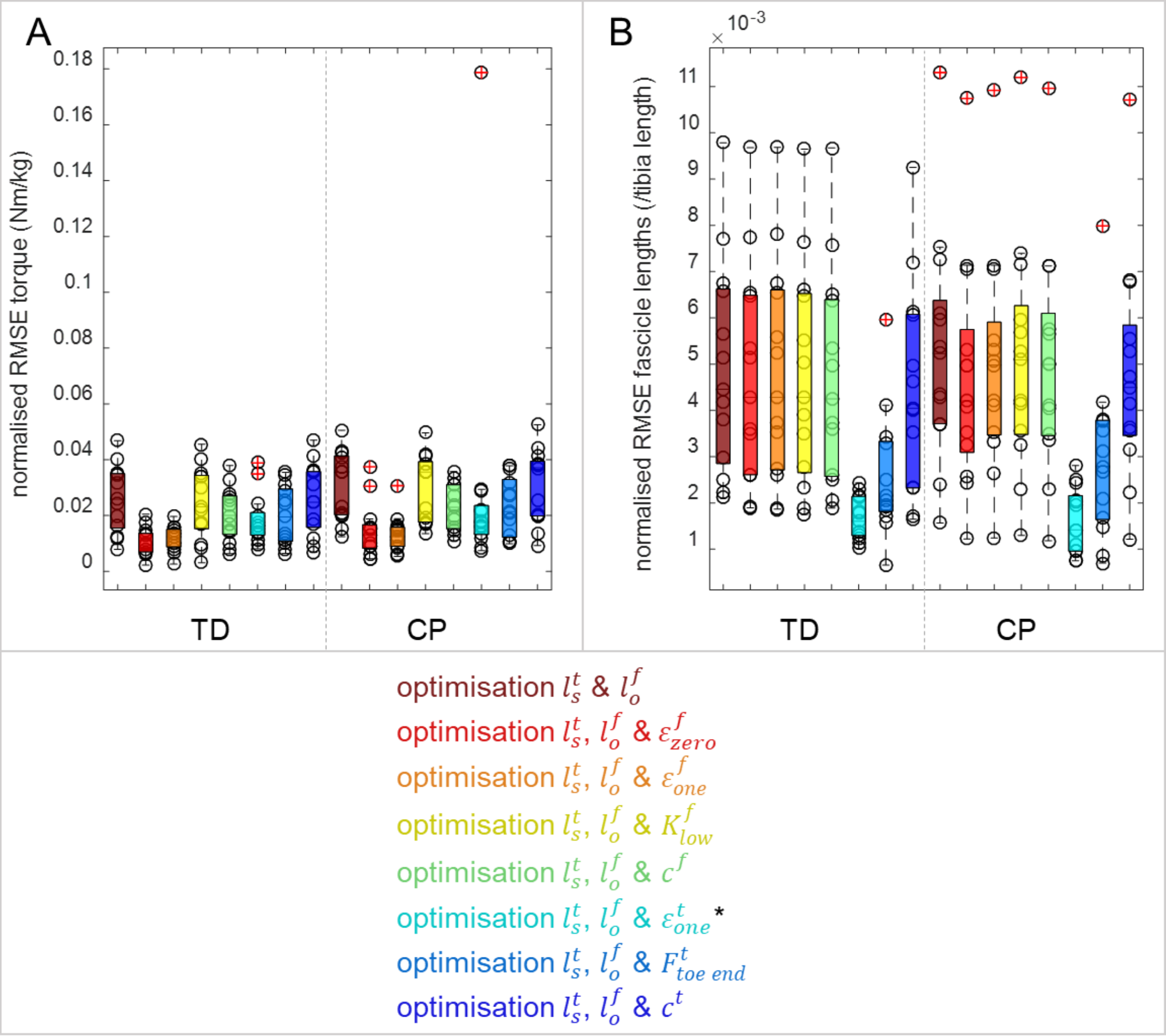

Optimisation l_s^t, l_o^f & ε_{one}^t

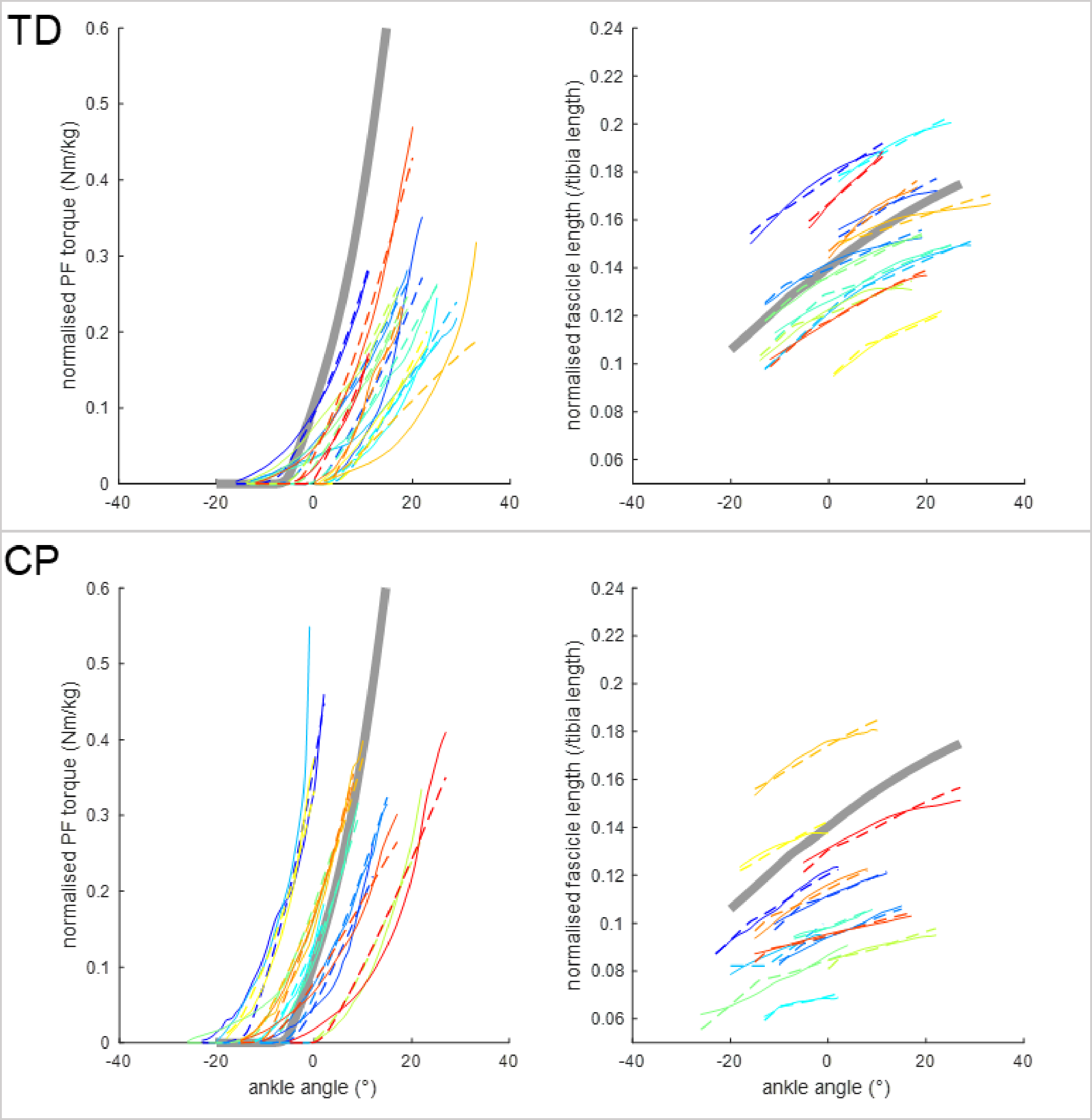

Individual measured (solid coloured) and optimised (l_s^t, l_o^f & ε_{one}^t; dashed coloured lines), and default (grey lines) plantarflexor (PF) torque-angle (left) and fascicle length-angle (right) curves for TD and CP. Each colour indicates a different individual.

Step 4: $l_s^t, l_o^f, \varepsilon_{one}^t$ and each parameter

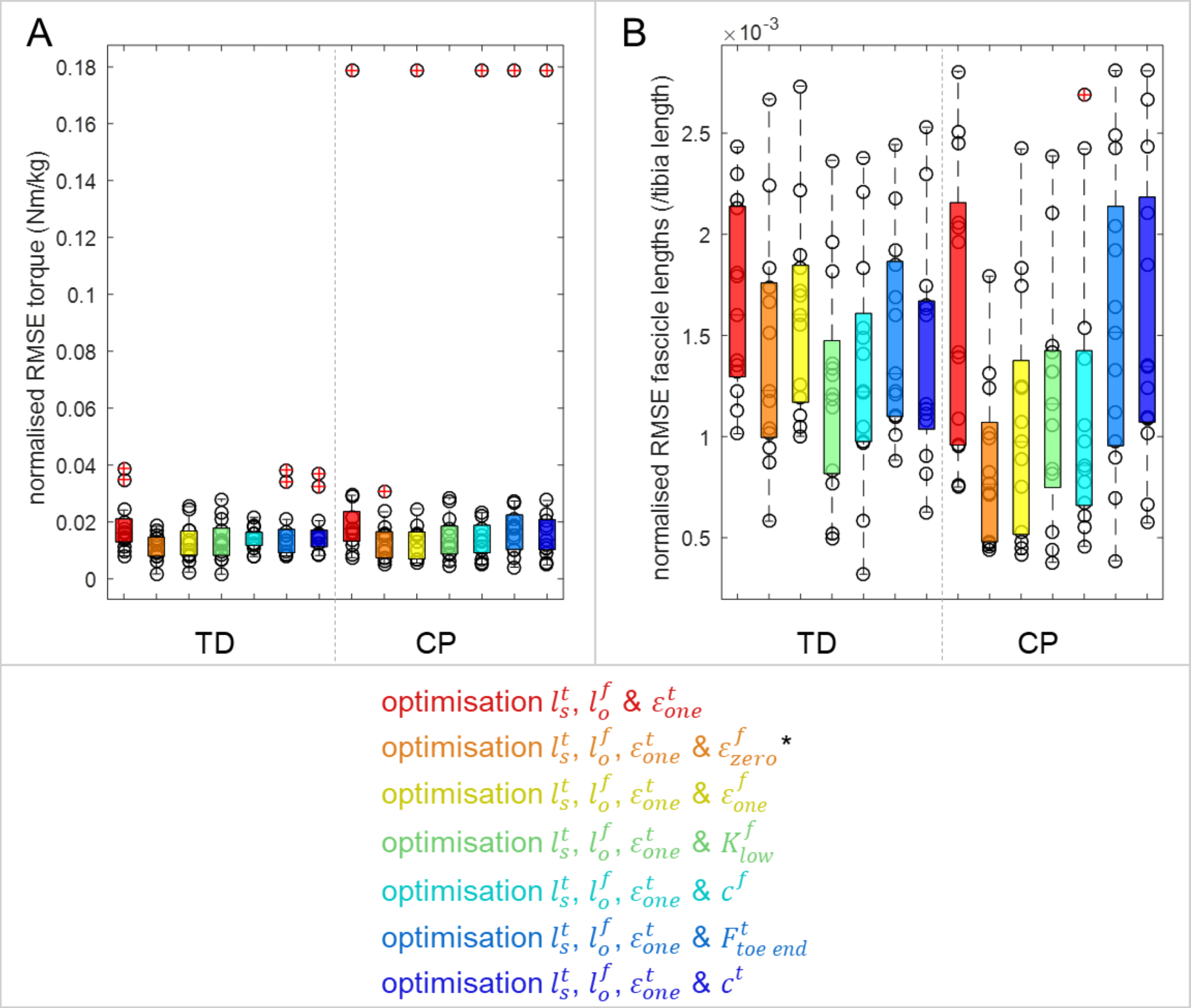

Optimisation l_s^t, l_o^f, ε_{one}^t & ε_{zero}^f

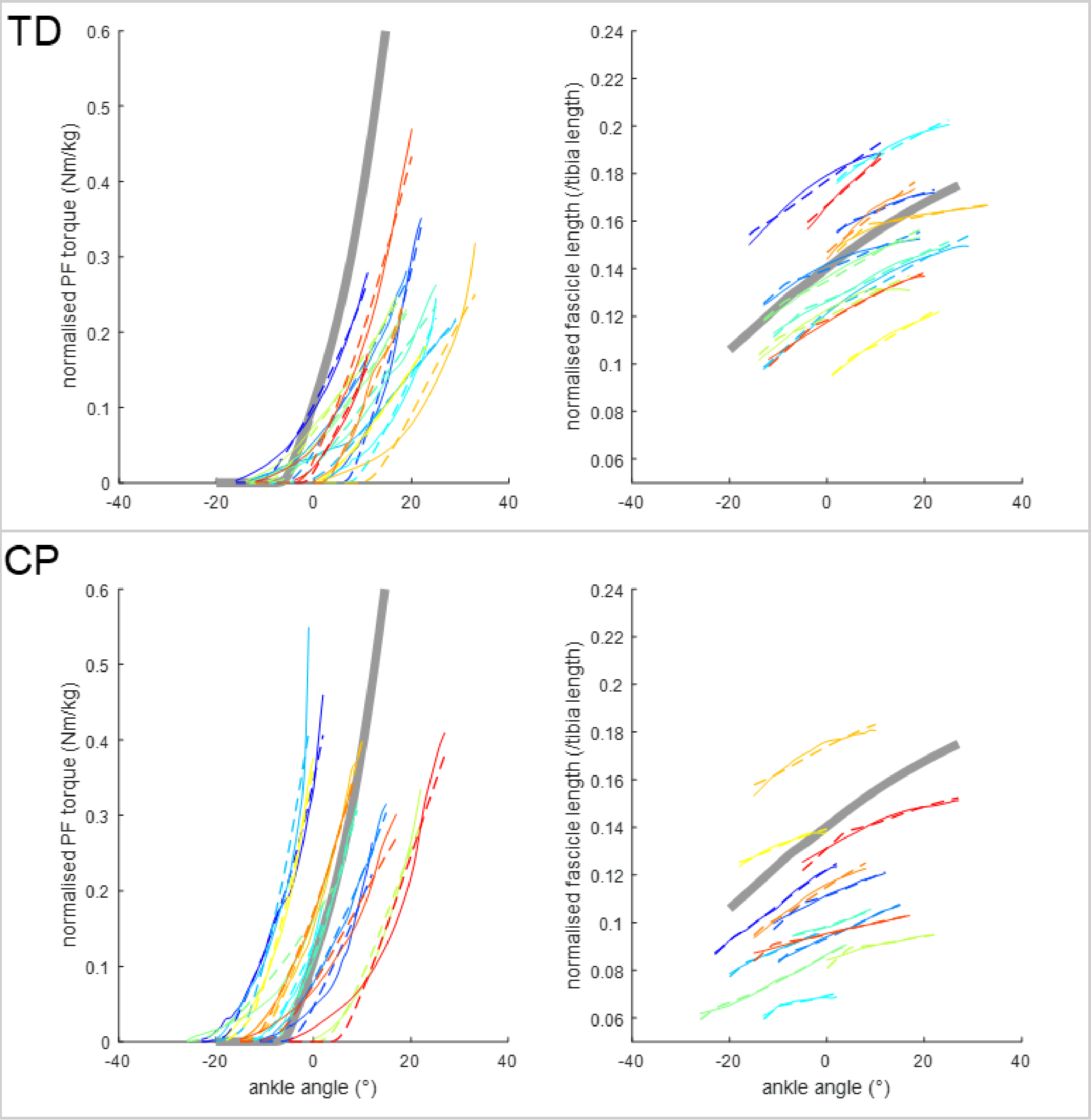

Individual measured (solid coloured) and optimised (l_s^t, l_o^f, ε_{one}^t & ε_{zero}^f; dashed coloured lines), and default (grey lines) plantarflexor (PF) torque-angle (left) and fascicle length-angle (right) curves for TD and CP. Each colour indicates a different individual.

Appendix B: Check for parameter redundancy

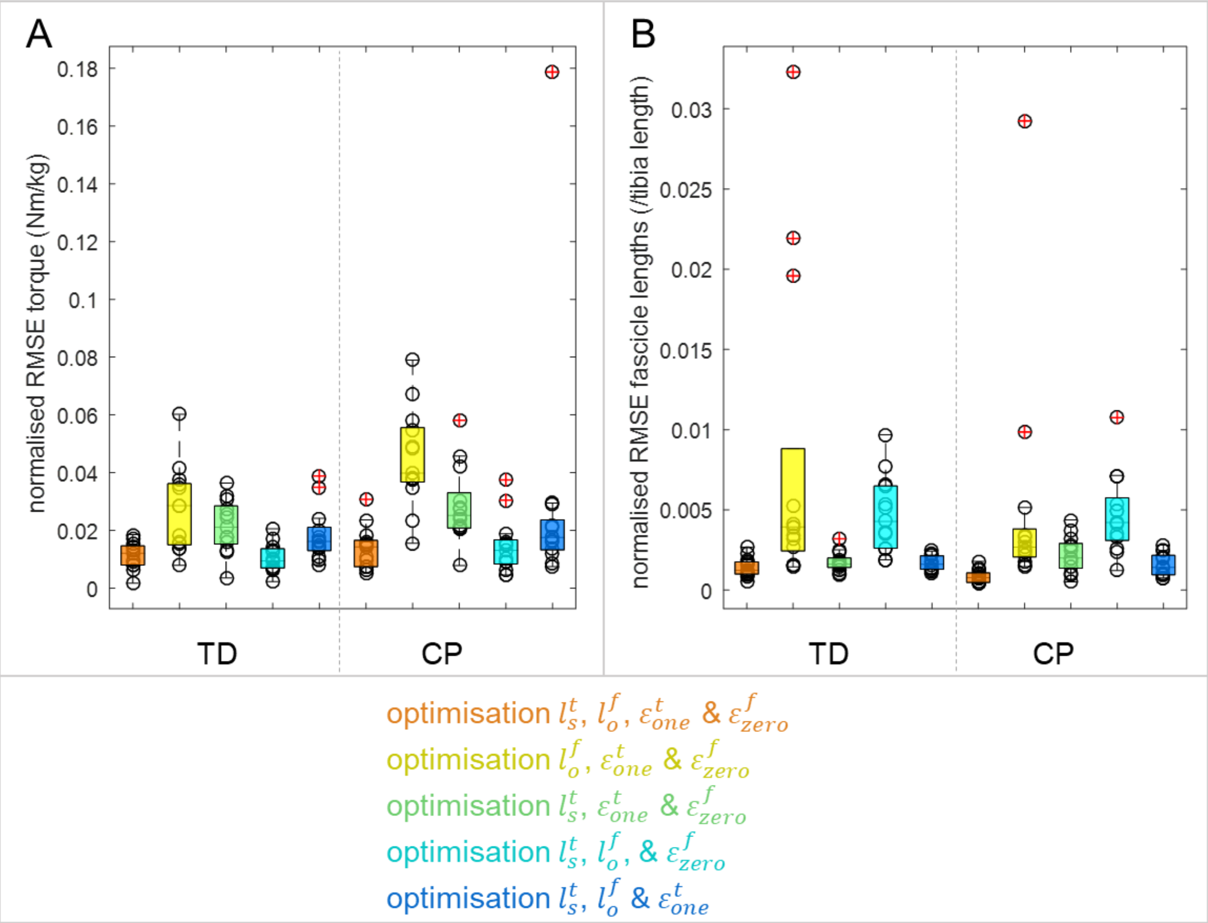

Supplement: Supplementary file 1 — Supplementary file1 (PDF 1684 kb) [file 10439_2022_3107_MOESM1_ESM.pdf]
